# Supplementary material for: Inflammatory responses in SARS-CoV-2 associated Multisystem Inflammatory Syndrome and Kawasaki Disease in children: An observational study
Source: PLoS One. 2022 Nov 30;17(11):e0266336. doi: 10.1371/journal.pone.0266336 (PMC9710748; doi:10.1371/journal.pone.0266336)
Supplement: S1 File — (PDF) [file pone.0266336.s001.pdf]

S1 File: Supplemental Methods

MIS-C criteria:

All MIS-C patients met the CDC or WHO criteria for MIS-C , *i.e.*: (1) age <21 years presenting with fever (>38.0°C) for ≥ 48 hours, or history of fever lasting ≥ 24 hours), laboratory evidence of inflammation, and evidence of multisystem (≥2) organ involvement (cardiac, renal, respiratory, hematologic, gastrointestinal, dermatologic and/or neurological), and (2) no alternative plausible diagnoses (in retrospect), and (3) positive test results for SARS-CoV-2 infection by RT-PCR or positive systemic SARS-CoV-2 specific total antibodies, or documented SARS-CoV-2 exposure within four weeks prior to onset of symptoms. Laboratory markers for inflammation consisted of, but were not limited to, one or more of the following: elevated C-reactive protein (CRP), erythrocyte sedimentation rate (ESR), or ferritin (supplemental table S1).

SARS-CoV-2 antibody titers

In all patients, circulating total antibodies (combined IgA, IgM and IgG) and IgM antibodies to SARS-CoV-2 were determined using the SARS-CoV-2 total antibody and the SARS-CoV-2 IgM assay (Wantai Biological Pharmacy Enterprise Co., Beijing, China), following the manufacturer's instructions. In five out of eight MIS-C patients, antibodies were measured over time (Table S1 and Figure S2).

Serum protein analysis

Cytokine levels and additional biomarkers in serum were measured with the Bio-Plex system and analyzed with the BioPlex Manager software version 6.2 (Bio-rad Laboratories, Hercules CA, USA) which uses the Luminex xMap technology according to the manufacturer's protocols. A total of 105 unique serum proteins were measured. Proteins for which more than 2/3 (66%) of measurements were below the detectable range were excluded for further analysis (IL-27-A, IFN-alpha-2, IFN-lambda-1, IL-12 subunit p70, IL-13, IL-17A, IL-22, IL-34, IL-35, MMP-1). 89 serum proteins were available for analysis (APRIL, BAFF, Beta-NGF, CCL1, CCL11, CCL13, CCL15, CCL17, CCL19, CCL2, CCL20, CCL21, CCL22, CCL23, CCL24, CCL25, CCL26, CCL27, CCL3, CCL7, CCL8, Chitinase-3-like protein 1, CXCL1, CXCL10, CXCL11, CXCL12, CXCL13, CXCL16, CXCL2, CXCL5, CXCL6, CXCL9, FGF-2, Fractalkine, G-CSF, GM-CSF, HGF, IFN-beta, IFN-gamma, IFN-lambda-2, IL-1 alpha, IL-1 beta, IL-10, IL-11, IL-12 subunit p40, IL-15, IL-16, IL-18, IL-19, IL-1ra, IL-2, IL-20, IL-26, IL-2-RA, IL-3, IL-32, IL-4, IL-5, IL-6, IL-6RB, IL-7, IL-8, IL-9, LIF, LIGHT, M-CSF, MIF, MIP-1-alpha, MIP-1-beta, MMP-2, MMP-3, Osteocalcin, Osteopontin, PDGF-BB, Pentraxin-related protein PTX3, RANTES, sCD163, sCD30, SCF, sIL-6RA, Stem Cell Growth Factor beta, sTNF-R1, sTNF-R2, TNF-a, TNF-b, TRAIL, TSLP, TWEAK, VEGF).

In vitro T-cell stimulation assay

Cryopreserved peripheral blood mononuclear cells (PBMCs) were isolated from heparinized blood samples using standard ficoll density gradient centrifugation. A minimum number of 1.5 - 2 million PBMCS were required for the stimulation experiments. The SARS-CoV-2 peptide pools consisted of 315 peptides (delivered in two subpools of 158 & 157 peptides) derived from a peptide scan (15mers with 11 aa overlap) through Spike glycoprotein of SARS-CoV-2 (product code PM-WCPV-S-2). Cells were incubated with peptide pools at 37°C (in 5% CO_2). Unstimulated cells, with no added peptides, were used a negative control, and a CMV pp65 peptide pool (100 ng/mL, PepTivator CMV pp65, Miltenyi) was used for comparison. Activation with soluble αCD3 (Thermofisher, clone HIT3A) was used as a positive control and unstimulated cells (no addition of peptides) where used as a negative control. After two hours, Brefeldin A (Thermofisher) was added and cells were further incubated at 37º C (in 5% CO_2) overnight.

Flow cytometric analyses

PBMCs were stained with combinations of the following antibodies: anti-CD3 BUV661 (BD, clone UCHT1), anti-CD4 BUV737 (BD, clone SK3), anti-CD8 BUV805 (BD, clone SK1), anti-CD45RA BUV563 (BD, clone HI100), anti-CD27 BV650 (Biolegend, clone O323), anti-CD40L PeDazzle594 (Biolegend, clone 24–31), anti-IFN- γ BV785 (Biolegend, clone 4S.B3).

Cells were stained according to the manufacturer's instructions and analyzed in PBS 0.5% FCS. For intracellular staining of IFN-γ, cells were fixed and permeabilized with the Foxp3 Staining Buffer Set (Thermofisher). Samples were acquired on a BD FACSymphony. Data analysis was performed using FlowJo (TreeStar, Version 10.0.7) and adhere to the Guidelines for the use of flow cytometry and cell sorting in immunological studies
