# Supplementary material for: Inflammatory responses in SARS-CoV-2 associated Multisystem Inflammatory Syndrome and Kawasaki Disease in children: An observational study
Source: PLoS One. 2022 Nov 30;17(11):e0266336. doi: 10.1371/journal.pone.0266336 (PMC9710748; doi:10.1371/journal.pone.0266336)
Supplement: S1 Table — (PDF) [file pone.0266336.s002.pdf]

S1 Table: Patients characteristics

	Patient no.	1	2	3	4	5	6	7	8	9	10	11	12	13	14
**Demographics**	**Age**	17	17	7	11	15	16	2	7	19	3	3	9	11	11
	**Sex**	F	F	F	M	F	F	M	F	M	F	M	M	M	F
	**Etnicity**	Cauc.	non-cauc.	non-cauc.	Cauc.	non-cauc.	Cauc.	Cauc.	Cauc.	Cauc.	Cauc.	non-cauc.	Cauc.	non-cauc.	non-cauc.
	**BMI**	20,2	29,1	18,0	15,2	21.6	22,7	14,1	18,2	28,3	17,6	17,0	15,9	22,4	24,0
**Classification**	**MIS-C[c]**	+	+	+	+	+	-	-	-	+	-	-	-	+	+
	**Kawasaki Disease[b]**	-	±	+	+	±	+	±	±	+	+	+	+	-	-
	**Signs of MAS[e]**	+	+	-	+	-	-	-	-	+	-	-	-	-	-
	**Shock[a]**	+	+	-	-	+	+	+	-	+	-	-	-	-	+
**Signs& Symptoms**															
	**Respiratory Distress**	+	+	+	-	+	-	+	-	-	-	-	-	+	n/a
	**Fever > 5 days[d]**	-	+	+	+	+	+	+	+	+	+	+	+	+	+
	**Rash**	-	+	+	+	+	+	+	+	+	+	-	+	-	-
	**Erythema of Palms/Soles**	-	+	+	+	+	+	+	+	+	+	-	+	-	-
	**Conjunctivitis**	-	+	+	+	+	+	-	-	+	+	+	-	-	+
	**Mucositis**	-	+	+	+	-	+	-	-	+	+	+	+	-	-
	**Lymphadenopathy**	-	-	+	+	-	-	-	+	-	+	+	+	-	-
	**Abdominal Symptoms**	+	+	+	+	+	+	+	+	+	-	-	+	-	+
	**Acute Kidney Injury**	+	+	-	-	+	+	+	-	+	-	-	-	-	-
	**Neurological Symptoms**	+	-	-	-	-	-	-	-	-	-	-	-	-	-
**SARS-CoV-2**	**PCR (Nasoph.)**	-	-	-	-	-	-	-	-	+	-	-	-	-	n/a
	**PCR (Feces)**	n/a	n/a	n/a	-	-	n/a	-	n/a	-	-	n/a	-	+	n/a
	**History of Contact**	-	+	-	+	+	-	-	-	+	-	-	-	-	-
	**Serology**	+	+	+	+	+	-	-	-	+	-	-	n/a	+	+
	**1st Serology taken @symptoms**	10	13	5	11	7	63	46	23	15	4	n/a	n/a	n/a	n/a
	**IgG**	+	+	+	+	+	-	-	-	+	-	-	-	+	+
	**IgM**	+	+	+	n/a	+	-	-	-	+	-	-	-	n/a	+
	**2nd Serology taken @symptoms**	68	49	49	48	n/a	n/a	n/a	n/a	39	n/a	n/a	n/a	n/a	n/a
	**IgG**	+	+	+	+	n/a	n/a	n/a	n/a	+	n/a	n/a	n/a	+	+
	**IgM**	+	-	-	+	n/a	n/a	n/a	n/a	+	n/a	n/a	n/a	+	-
**Cardiac Markers and Echo 1 & 2**	**Troponin (ng/l)**	n/a	438	n/a	n/a	89	n/a	85	n/a	378	n/a	n/a	n/a	774	115
	**Nt-Pro BNP (ng/l)**	n/a	30848	n/a	n/a	n/a	n/a	33798	n/a	n/a	99	n/a	n/a	6461	9320
	**1: Poor LV function**	-	+	-	-	+	n/a	+	n/a	-	-	-	-	-	-
	**1: Myocarditis**	-	+	-	-	+	n/a	-	n/a	-	-	-	-	-	-
	**1: Ejection Fraction (%)**	51	33	52	n/a	n/a	n/a	n/a	n/a	n/a	n/a	60	n/a	49	n/a

	**1: Prominent Coronary Arteries**	n/a	n/a	+ (z+2,7)	n/a	n/a	n/a	-	n/a	-	n/a	n/a	n/a	-	+ (Z+4)
	**Repeat US after (days)**	60	50	51	32	n/a	73	59	42	24	n/a	60	n/a	48	49
	**Patient no.**	1	2	3	4	5	6	7	8	9	10	11	12	13	14
**Cardiac Markers and Echo 1 & 2**	**2: Poor LV function**	-	-	-	-	n/a	-	-	-	-	n/a	-	n/a	-	-
	**2: Myocarditis**	-	-	-	-	n/a	-	-	-	-	n/a	-	n/a	-	-
	**2: Ejection Fraction (%)**	50	50	50	58	n/a	56,1	n/a	52	50	n/a	58,5	n/a	51,8	59,1
	**2: Shortening Fraction (%)**	39	34,5	32,1	32,7	n/a	37,4	32	30,3	35,2	n/a	40,5	n/a	39,7	44,4
	**2: Coronary Dilatation**	-	-	-	-	n/a	-	-	-	-	n/a	-	n/a	-	-
**Treatment & Outcome**	**IVIG**	-	-	+	+	+	-	-	-	+	+	+	-	+	+
	**Steroids**	+	-	-	-	+	-	-	-	+	+	-	-	+	+
	**Antibiotics**	+	+	+	+	+	+	-	+	+	-	-	+	-	+
	**Antivirals**	+	-	-	-	-	-	-	-	-	-	-	-	-	-
	**Oral Anticoagulation**	-	-	+	+	-	-	-	-	+	+	+	-	-	-
	**LWMH**	-	-	-	-	+	-	-	-	+	-	-	-	+	+
	**Inotropes**	+	+	-	-	+	+	+	-	+	-	-	-	+	+
	**Admission Days**	12	8	5	5	12	5	19	n/a	10	7	5	n/a	n/a	n/a
	**PICU Admission**	+	+	+	-	+	+	+	-	+	-	-	-	+	+
	**Death**	-	-	-	-	-	-	-	-	-	-	-	-	-	-
**Laboratory Results**	**Hb (g/dl)**	11.1	11.3	6.4	12,7	13.8	10.3	15,9	13.5	15,9	11.6	11.1	12.7	n/a	n/a
	**ESR (mm/h)**	n/a	n/a	n/a	33	140	n/a	10	54	n/a	43	87	5	34	n/a
	**CRP (mg/l)**	270	404	435	300	506	40	191	54	466	169	27	65	114	336
	**Hb (mmol/l)**	6,9	7	4	7,9	8,6	6,4	9,9	8,4	9,9	7,2	6,9	7,9	n/a	n/a
	**Lymphocytes (x10e9/l)**	4,5	1,21	2	0,58	0,49	0,15	3,2	5,2	0,66	1,4	0,17	0,5	0,46	5
	**Neutrophils (x10e9/l)**	12,5	12,96	9,8	7,7	15,6	14,5	1,2	6,1	12,5	7	8,99	6,6	5,66	9,2
	**Platelets (x10e9/l)**	114	114	590	126	250	81	346	285	190	210	230	214	181	150
	**Albumin (g/l)**	n/a	n/a	25	38	35	24	17	n/a	21	23	n/a	44	39	27
	**Ferritin (ug/L)**	1066	1141	352	4729	663	n/a	1278	79	3350	104	214	80	1504	979
	**ASAT (IU/L)**	58	61	28	151	21	58	156	33	39	29	78	38	40	52
	**Triglycerides (mmol/l)**	240	259	n/a	193	151	n/a	23	96	195	n/a	n/a	n/a	127	407
	**Fibrinogen (g/l)**	700	n/a	n/a	n/a	1200	460	390	n/a	270	n/a	n/a	n/a	n/a	n/a
	**D-Dimers (mg/l)**	n/a	n/a	n/a	n/a	3,26	n/a	n/a	n/a	n/a	n/a	n/a	n/a	7,84	2,17
	**Creatinine (umol/l)**	450	405	34	56	113	285	49	47	132	20	22	41	36	124
**Microbiology**	**Culture Blood**	-	-	-	-	-	-	-	-	-	-	-	n/a	n/a	n/a
	**Culture: Urine**	*E.coli*	-	-	n/a	n/a	n/a	-	n/a	-	-	-	n/a	n/a	n/a
	**Culture: Stool**	-	-	n/a	-	n/a	-	-	n/a	-	-	n/a	n/a	n/a	n/a
	**Culture: Sputum**	n/a	n/a	n/a	n/a	n/a	n/a	n/a	n/a	n/a	-	n/a	n/a	n/a	n/a
	**PCR: Atypitcal Resp. Pathogens**	-	-	n/a	n/a	-	-	n/a	-	-	n/a	n/a	n/a	n/a	n/a

**PCR: Resp. Virus**	-	-	-	n/a	-	-	n/a	-	n/a	n/a	-	-	n/a	n/a
**PCR: Stool Viral**	-	-	-	-	-	-	-	n/a	n/a	-	n/a	-	n/a	n/a
**PCR: CMV Blood**	-	-	-	n/a	n/a	n/a	n/a	n/a	n/a	n/a	n/a	n/a	n/a	n/a
**PCR: EBV Blood**	-	n/a	-	n/a	n/a	n/a	n/a	n/a	n/a	n/a	n/a	n/a	n/a	n/a

n/a missing data

± incomplete Kawasaki Disease

a. Shock defined as needing inotropic support or fluid resuscitation >20 ml/kg

b. American Heart Association criteria for the definition of Kawasaki disease (KD) is to have persistent fever and 4 of the following 5 mucocutaneous features: erythema and cracking of lips, strawberry tongue, and/or erythema of oral and pharyngeal mucosa; bilateral bulbar conjunctival injection without exudate; rash (maculopapular, diffuse erythroderma); erythema and edema of the hands and feet in acute phase and/or periungual desquamation in subacute phase; and cervical lymphadenopathy (>1.5 cm diameter). Incomplete KD was defined by at least 2 clinical criteria compatible with KD and additional laboratory or cardiac criteria.

c. MIS-C according to criteria by Centers for Disease Control and Prevention (CDC) and World Health Organization (WHO)

d. Fever >38 °C

e. Case definition for macrophage activation syndrome; fever; and ferritin>684 ng/ml, and 2 of the following criteria: platelets <181 x 10^9/L, AST >48 IU/L, triglycerides > 156 mg/dl or fibrinogen < 360 mg/dl
