# Supplementary material for: Inflammatory responses in SARS-CoV-2 associated Multisystem Inflammatory Syndrome and Kawasaki Disease in children: An observational study
Source: PLoS One. 2022 Nov 30;17(11):e0266336. doi: 10.1371/journal.pone.0266336 (PMC9710748; doi:10.1371/journal.pone.0266336)
Supplement: S2 Table — (PDF) [file pone.0266336.s003.pdf]

S2 Table Serum proteins levels, 2Log fold expression compared to healthy controls.

	Mann-Whitney U	Correction for multiple testing		2Log fold compared to geometric mean of HD			
				SARS neg		SARS pos	
**Serum protein**	**P-value (2-tailed)**	**Q-value**	**Discovery?**	**AP**	**FU**	**AP**	**FU**
**IL-2-RA**	0.00001	0.00006	Yes	2.37	1.80	3.63	1.83
**M-CSF**	0.00001	0.00006	Yes	4.18	3.46	4.40	2.65
**Beta-NGF**	0.00002	0.00006	Yes	1.51	1.35	1.59	1.39
**HGF**	0.00002	0.00006	Yes	4.53	2.09	4.79	2.15
**IL-1ra**	0.00002	0.00006	Yes	3.80	3.11	4.57	2.96
**IL-6**	0.00002	0.00006	Yes	4.55	2.12	4.91	3.14
**sCD163**	0.00002	0.00006	Yes	2.87	1.15	3.08	1.56
**sTNF-R1**	0.00002	0.00006	Yes	3.24	1.49	3.56	1.85
**sTNF-R2**	0.00002	0.00006	Yes	3.85	2.11	3.83	2.22
**BAFF**	0.00003	0.00006	Yes	2.47	0.75	3.59	1.49
**IL-1 beta**	0.00003	0.00006	Yes	2.07	1.85	2.24	1.79
**IL-18**	0.00003	0.00006	Yes	4.83	5.05	5.92	3.69
**TSLP**	0.00003	0.00006	Yes	2.76	1.84	2.60	1.90
**IL-16**	0.00004	0.00007	Yes	3.27	3.90	2.89	2.42
**Pentraxin-related protein PTX3**	0.00004	0.00007	Yes	3.65	2.64	3.98	3.17
**G-CSF**	0.00005	0.00007	Yes	2.86	1.06	3.48	1.25
**IL-11**	0.00005	0.00007	Yes	1.78	0.96	1.71	1.39
**MIP-1-alpha**	0.00005	0.00007	Yes	3.90	1.35	4.53	1.89
**sCD30**	0.00005	0.00007	Yes	3.26	2.78	3.38	1.72
**IL-8**	0.00006	0.00008	Yes	3.99	2.87	3.61	2.20
**MIP-1-beta**	0.00006	0.00008	Yes	1.44	1.47	1.69	1.22
**IFN-beta**	0.00007	0.00008	Yes	2.14	0.64	2.19	0.83
**IL-2**	0.00007	0.00008	Yes	1.50	1.49	1.77	1.07
**CCL3**	0.00011	0.00012	Yes	2.86	0.88	2.96	1.23
**CXCL16**	0.00011	0.00012	Yes	1.05	0.06	0.61	0.14
**Stem Cell Growth Factor beta**	0.00011	0.00012	Yes	1.27	0.14	1.62	0.93
**SCF**	0.00013	0.00013	Yes	2.10	2.12	1.96	1.50
**CCL15**	0.00014	0.00014	Yes	1.80	0.63	1.22	0.60
**CXCL5**	0.00015	0.00014	Yes	2.81	2.79	2.73	2.83
**CCL2**	0.00017	0.00015	Yes	1.39	1.02	2.21	1.19
**CCL25**	0.00017	0.00015	Yes	2.03	1.51	1.45	1.16
**CCL27**	0.00017	0.00015	Yes	1.24	0.99	1.54	0.42
**Chitinase-3-like protein 1**	0.00022	0.00018	Yes	2.14	0.58	2.28	0.55
**CXCL10**	0.00033	0.00026	Yes	1.45	-0.58	4.71	1.14
**GM-CSF**	0.00033	0.00026	Yes	1.96	2.16	3.10	2.26
**IL-5**	0.00039	0.00030	Yes	1.85	0.32	1.85	0.79
**TWEAK**	0.00041	0.00030	Yes	2.27	3.00	1.89	1.99
**IFN-gamma**	0.00045	0.00032	Yes	1.25	0.82	3.70	1.90
**MMP-3**	0.00049	0.00033	Yes	2.36	0.88	2.30	1.67
**Osteopontin**	0.00049	0.00033	Yes	3.65	3.07	2.15	3.40
**TNF-a**	0.00050	0.00033	Yes	2.28	1.48	2.19	1.61
**IL-10**	0.00064	0.00042	Yes	1.52	0.90	1.68	0.71
**FGF-2**	0.00088	0.00055	Yes	0.95	0.65	0.86	0.81
**IL-3**	0.00090	0.00055	Yes	1.00	0.52	1.17	0.74
**CCL19**	0.00091	0.00055	Yes	2.10	1.94	2.76	1.74
**MIF**	0.00110	0.00065	Yes	3.15	3.71	2.32	3.55

Serum protein	Mann-Whitney U P-value (2-tailed)	Correction for multiple testing Q-value	Discovery?	2Log fold compared to geometric mean of HD SARS neg AP	FU	SARS pos AP	FU
**IL-12 subunit p40**	0.00155	0.00090	Yes	1.31	0.40	1.02	0.50
**IL-20**	0.00166	0.00094	Yes	1.76	0.97	1.54	0.68
**IL-15**	0.00175	0.00097	Yes	0.86	0.51	0.28	0.68
**IL-19**	0.00192	0.00103	Yes	1.12	0.82	1.17	0.48
**CXCL11**	0.00193	0.00103	Yes	1.68	1.18	4.79	2.71
**PDGF-BB**	0.00211	0.00111	Yes	1.21	1.11	2.03	1.15
**IL-1 alpha**	0.00216	0.00111	Yes	1.15	1.06	1.48	1.16
**CCL21**	0.00278	0.00135	Yes	1.03	1.49	1.84	1.75
**Fractalkine**	0.00278	0.00135	Yes	0.64	0.13	0.83	-0.01
**sIL-6RA**	0.00278	0.00135	Yes	1.68	1.07	1.22	0.57
**LIGHT**	0.00319	0.00153	Yes	1.41	-0.42	1.95	0.49
**CCL20**	0.00331	0.00156	Yes	2.77	0.43	3.00	1.43
**TRAIL**	0.00380	0.00176	Yes	2.23	2.12	2.20	2.31
**IFN-lambda-2**	0.00463	0.00206	Yes	1.46	0.88	1.00	0.56
**TNF-b**	0.00467	0.00206	Yes	0.52	0.94	1.02	0.97
**CCL11**	0.00468	0.00206	Yes	2.00	2.22	2.06	1.35
**CCL22**	0.00769	0.00328	Yes	-0.60	0.53	-1.03	-0.71
**CXCL9**	0.00769	0.00328	Yes	0.58	-0.66	3.31	0.39
**CCL7**	0.00881	0.00370	Yes	0.77	0.69	1.27	0.78
**CXCL1**	0.00901	0.00372	Yes	0.51	0.44	0.93	0.38
**CCL8**	0.01141	0.00464	Yes	0.12	-0.61	2.67	0.02
**IL-9**	0.01929	0.00774	Yes	0.30	0.93	1.03	1.02
**APRIL**	0.02234	0.00883	Yes	-0.20	-1.16	2.01	0.20
**IL-7**	0.02466	0.00961	Yes	0.57	0.86	0.94	1.64
**IL-4**	0.02955	0.01135	No	0.58	0.48	0.51	0.28
**VEGF**	0.03473	0.01315	No	1.01	0.19	0.86	0.74
**LIF**	0.04000	0.01494	No	0.68	0.90	0.87	0.77
**CXCL13**	0.05021	0.01850	No	0.52	0.04	1.05	0.11
**MMP-2**	0.05126	0.01864	No	2.16	2.54	1.43	1.10
**IL-32**	0.05913	0.02122	No	1.21	0.17	0.33	0.26
**CCL26**	0.08170	0.02894	No	0.95	0.92	0.64	0.46
**CXCL12**	0.12769	0.04410	No	-0.34	-0.42	0.76	-0.04
**CCL23**	0.12776	0.04410	No	1.13	-0.60	0.58	-0.26
**IL-6RB**	0.14194	0.04838	No	0.40	0.15	0.25	-0.15
**IL-26**	0.15918	0.05359	No	1.03	1.09	-0.03	0.72
**RANTES**	0.17389	0.05783	No	-1.09	0.07	0.47	0.36
**CCL13**	0.25335	0.08324	No	-0.06	-0.37	0.76	-0.20
**CCL24**	0.30101	0.09772	No	-0.76	-0.01	0.21	-0.67
**Osteocalcin**	0.38405	0.12321	No	0.12	2.05	-0.62	0.46
**CXCL6**	0.62446	0.19801	No	-0.46	-0.11	-0.11	-0.08
**CXCL2**	0.74392	0.23318	No	0.11	0.18	1.24	0.81
**CCL17**	0.78561	0.24345	No	0.04	0.75	0.22	-0.11
**CCL1**	0.95651	0.29308	No	0.26	0.26	0.52	0.32
