# Supplementary figures and images for: Inflammatory responses in SARS-CoV-2 associated Multisystem Inflammatory Syndrome and Kawasaki Disease in children: An observational study
Source: PLoS One. 2022 Nov 30;17(11):e0266336. doi: 10.1371/journal.pone.0266336 (PMC9710748; doi:10.1371/journal.pone.0266336)

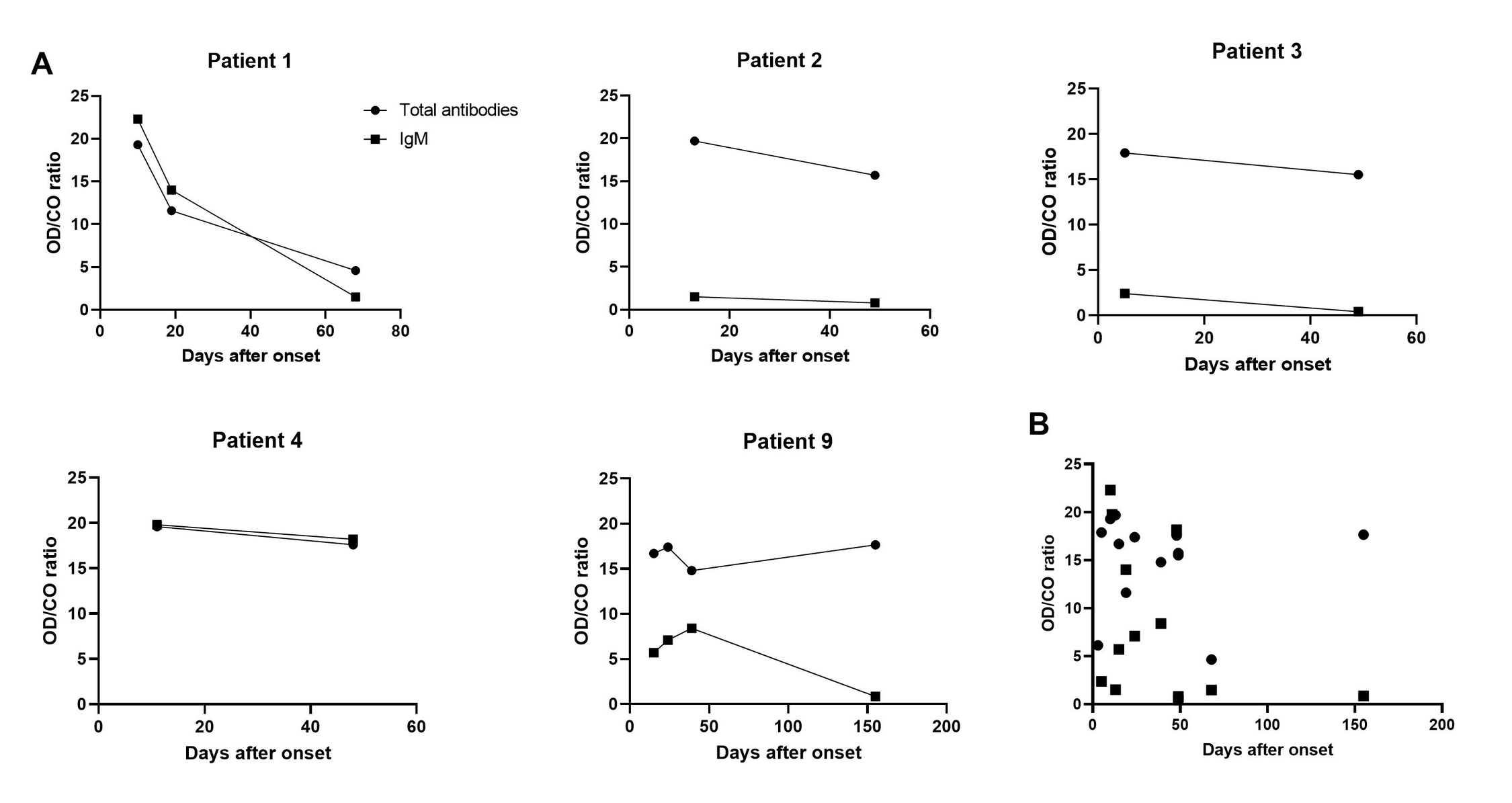

Supplement: S1 Fig — (A) Total (IgA, IgM, IgG) and IgM Spike-protein specific antibody titers measured in serum and calculated in OD/CO ratios over time in 5 MIS-C patients. (B) Total antibody titers in the MIS-C patients in relation to the time serum was obtained after admission. Antibodies were considered positive if the OD/CO ratio was > 1. Abbreviations: OD = optical density; the read out value of the antibody assay signal, CO = cut off; the threshold value for an antibody signal to be ‘positive’. (TIF) [file pone.0266336.s007.tif]

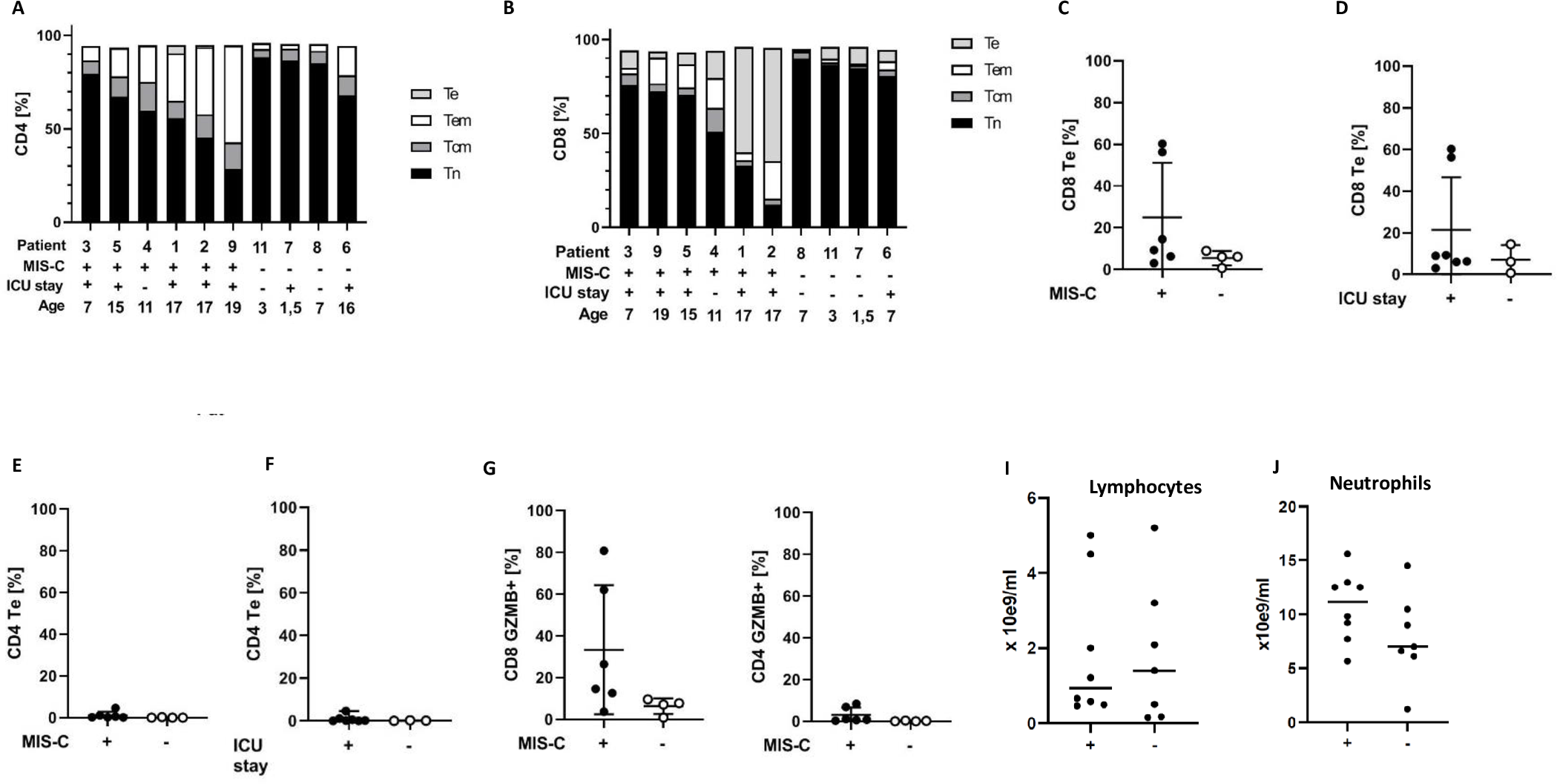

Supplement: S2 Fig — (A) C3+CD4+ subset T-cell counts depicted per patient, MIS-C, ICU stay and age. (B) C3+CD8+ subset T-cell counts depicted per patient, MIS-C, ICU stay and age. (C) CD3+CD8+ effector T-cell percentages in MIS-C (+) and KD patients (-). (D) CD3+CD8+ effector T-cell percentages depicted for patients that stayed at the ICU (+) or not (-). (E) CD3+CD4+ effector T-cell percentages in MIS-C (+) and KD patients (-). (F) CD3+CD4+ effector T-cell percentages depicted for patients that stayed at the ICU (+) or not (-). (G) percentage of CD3+CD8+ GZMB positive T-cells (cytotoxic T-cells) in MIS-C (+) and KD patients (-). (H) Percentage of CD3+CD4+ GZMB positive T-cells in MIS-C (+) and KDe patients (-). (I) Total lymphocytes counts in MIS-C (+) and KD patients (-). (J) Total neutrophil counts in MIS-C (+) and KD patients (-). Abbreviations: Te = effector T-cells, Tem = effector memory T-cells, Tcm = central memory T-cells, Tn = naïve T-cells, GZMB = Granzyme B. (TIF) [file pone.0266336.s008.tif]

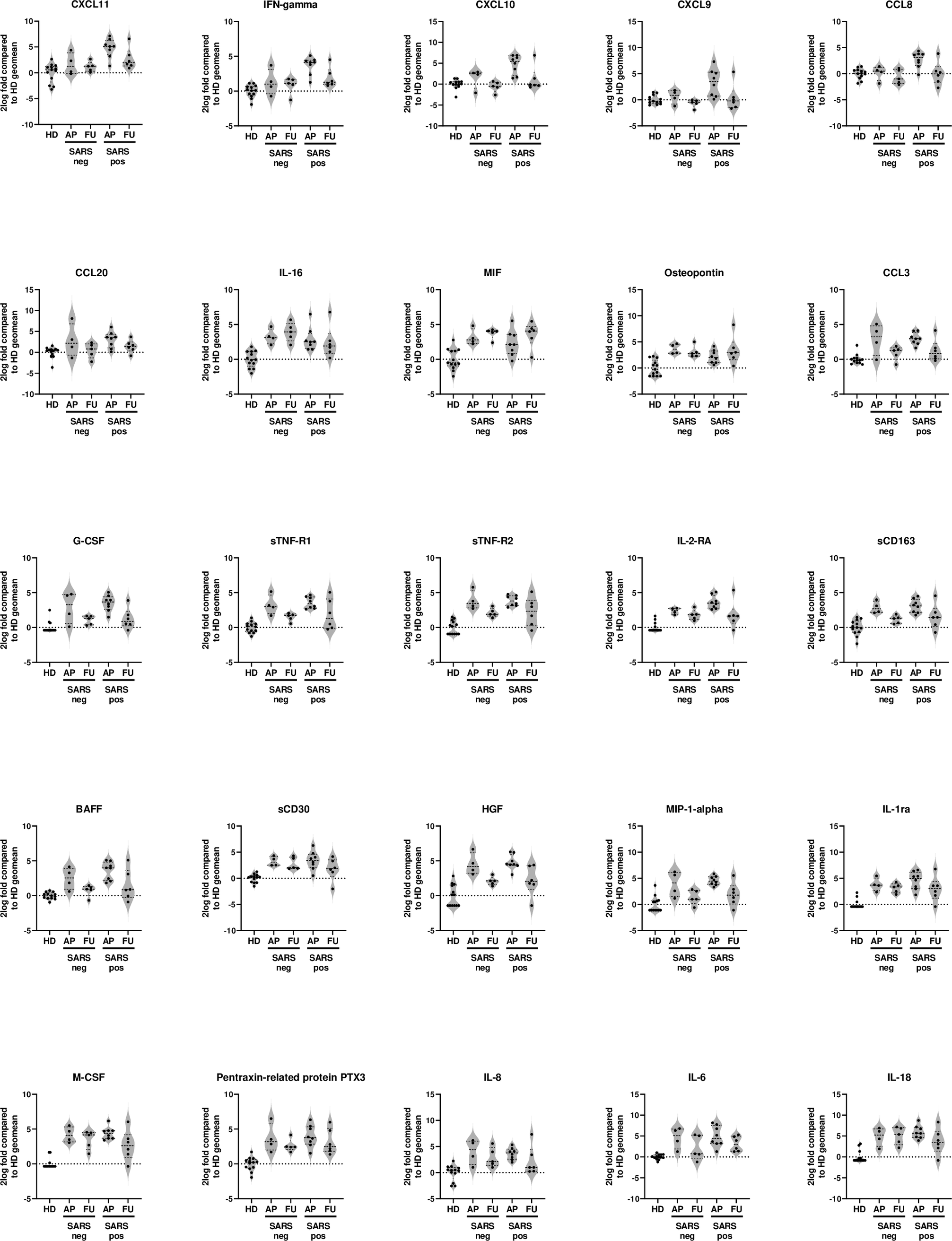

Supplement: S3 Fig — Violin plots of the highest log fold increased serum cytokines compared to healthy controls, and all represented in the lowest hierarchical cluster in Fig 2. (TIF) [file pone.0266336.s009.tif]
